# Pre-flight evaluation of adult patients with cystic fibrosis: a cross-sectional study

**DOI:** 10.1186/s13104-017-2386-2

**Published:** 2017-02-06

**Authors:** Elisabeth Edvardsen, Aina Akerø, Ole Henning Skjønsberg, Bjørn Skrede

**Affiliations:** 1 Department of Pulmonary Medicine, Oslo University Hospital, Ullevål, Oslo, Norway; 2 Faculty of Medicine, University of Oslo, Oslo, Norway; 3 National Center for Cystic Fibrosis, Oslo University Hospital, Oslo, Norway; 4 Department of Sports Medicine, Norwegian School of Sport Sciences, Oslo, Norway

**Keywords:** Blood gas response, Cardiopulmonary exercise testing, Gas exchange, Hypoxia altitude simulation test, Pulmonary function

## Abstract

**Background:**

Air travel may imply a health hazard for patients with cystic fibrosis (CF) due to hypobaric environment in the aircraft cabin. The objective was to identify pre-flight variables, which might predict severe hypoxaemia in adult CF patients during air travel.

**Methods:**

Thirty adult CF-patients underwent pre-flight evaluation with spirometry, arterial oxygen tension (PaO_2_), pulse oximetry (SpO_2_) and cardiopulmonary exercise testing (CPET) at sea level (SL). The results were related to the PaO_2_ obtained during a hypoxia-altitude simulation test (HAST) in which a cabin altitude of 2438 m (8000 ft) was simulated by breathing 15.1% oxygen.

**Results:**

Four patients fulfilled the criteria for supplemental oxygen during air travel (PaO_2 HAST_ < 6.6 kPa). While walking slowly during HAST, another eleven patients dropped below PaO_2 HAST_ 6.6 kPa. Variables obtained during CPET (PaO_2 CPET_, SpO_2 CPET_, minute ventilation/carbon dioxide output, maximal oxygen uptake) showed the strongest correlation to PaO_2 HAST_.

**Conclusions:**

Exercise testing might be of value for predicting in-flight hypoxaemia and thus the need for supplemental oxygen during air travel in CF patients.

*Trial registration* The study is retrospectively listed in the ClinicalTrials.gov Protocol Registration System: NCT01569880 (date; 30/3/2012)

## Background

Due to medical improvement and preventive initiatives, both quality of life and the longevity of patients with cystic fibrosis (CF) have increased substantially during the past 10–20 years. Today, CF-patients participate in all aspects of modern life, including air travelling. However, due to the hypobaric environment in the aircraft cabin, air travel may imply a health hazard for CF-patients, most of them suffering from chronic pulmonary disease. The ambient oxygen partial pressure inside the aircraft cabin during commercial air travel is reduced, corresponding to an altitude of 2438 m (8000 ft) at maximal cruising altitude [1]. CF-patients are therefore prone to a substantial reduction in the partial arterial oxygen pressure (PaO_2_) during the flight.

Guidelines concerning pre-flight evaluation of patients with pulmonary disease have suggested various screening variables as normative, such as oxygen saturation at sea level and spirometric values [2–4]. In case of doubt, a hypoxia-altitude simulation test (HAST) has been recommended as decisive. HAST is performed by breathing a gas mixture containing 15.1% oxygen corresponding to an altitude of 2438 metres [5]. Administration of supplemental oxygen during flight is advised if PaO_2_ drops below 6.6 kPa during the test [3, 4].

Several studies addressing pre-flight evaluation of lung patients have been published, mainly on patients with chronic obstructive pulmonary disease (COPD) [6–11]. However, these results may not be applicable to the CF-population. CF-patients are generally younger, have fewer additional (cardiovascular) risk factors, and the pathophysiology causing hypoxaemia may differ from that of COPD [12]. The literature regarding in-flight hypoxaemia in patients with CF is limited [13–18], and reliable screening methods for predicting development of severe in-flight hypoxaemia in CF patients have not been identified [3]. In COPD patients, it has been claimed that pre-flight measurement of arterial blood gases (PaO_2_) is the single most helpful test for predicting in-flight PaO_2_ in lung patients [19]. In previous reports on CF patients, however, arterial blood gas measurements have, to our knowledge, not been performed. In addition, recent studies have advocated that exercise testing is of value for predicting which patients will develop in-flight hypoxaemia [20, 21], but this aspect is also lacking in studies on CF-patients.

Given the widespread use of air travelling in adult patients with CF [16], further knowledge is needed to identify pre-flight variables which might predict in-flight hypoxaemia. The aims of the present study were to investigate the relationship between hypoxaemia achieved during HAST (PaO_2 HAST_) and sea level values of pulmonary function, arterial blood gases, pulse oximetry and cardiopulmonary exercise test (CPET) variables. In addition, the effect of slow walking during hypoxic exposure, simulating slow walking along the aircraft’s aisle, was studied.

## Methods

From January 2006 to December 2008, all adult CF patients referred to the National Center for Cystic Fibrosis at Oslo University Hospital, Norway for comprehensive assessment, and who were in a stable phase of their disease, were invited to participate in this cross-sectional HAST study. Criteria for eligibility were >16 years of age and sputum test negative for *Burkholderia cepacia* and/or methicillin resistant *Staphylococcus aureus*.

The CF diagnosis was confirmed both genetically and clinically. The Regional Committee for Medical Ethics (Oslo, Norway) approved the study, and written informed consents were obtained by all participants. The study is retrospectively listed in the ClinicalTrials.gov Protocol Registration System (NCT01569880).

### Pre-flight tests

Pre-flight tests were obtained at sea level (30 m above sea level, hereafter referred to as sea level) while breathing ambient air. Lung function tests were performed using Vmax Series, (VIASYS, Yorba Linda, USA) [22]. After 5 min at rest in the sitting position, the percutaneous oxygen saturation (SpO_2 SL_) was measured with a finger probe using a stationary pulse oximeter (NONIN 8600, Medical, Inc., Minneapolis, USA), and arterial blood gas samples were drawn from a radial artery cannula. The samples were immediately analysed (ABL 700 series, Radiometer, Copenhagen, Denmark).

CPET was performed by walking and running on a treadmill (Jaeger LE 100, Würzburg, Germany) until exhaustion, using a progressive 1 min step protocol. Gas exchanges and ventilatory variables were continuously sampled breath by breath through a Hans Rudolph two way breathing mask (Hans Rudolph Inc, Kansas City, USA). The breathing mask was connected to a Vmax SensorMedics metabolic analyzer (Yorba Linda, California, USA) to assess ventilatory variables and the content of oxygen and carbon dioxide of expired air for calculation of oxygen uptake (VO_2_). VO_2max_ was accepted when VO_2_ failed to raise despite rise in speed and ventilation (V_E_) or strong signs of exhaustion. Ventilation/perfusion mismatch was confirmed by impairment of ventilatory efficiency measured as elevated V_E_/VCO_2_ ratio at the anaerobic threshold calculated by the ventilatory equivalent method [23]. Arterial blood gases were sampled just before ending the exercise test.

### Simulated flight

HAST was performed while the participants were breathing a hypoxic gas mixture of 15.1% oxygen balanced with nitrogen via a non-diffusion gas collection bag (Hans Rudolph Inc., Kansas City, USA) in the sitting position for 15 min [5]. The bag was connected to a tight fitting mask with a two way non-breathing valve (Hans Rudolph Inc., Kansas City, USA). Subsequently, the participants walked on the treadmill at 1.2 km·hour^−1^ for 5 min corresponding to walking along the aisle, still breathing the hypoxic gas.

Arterial blood gases were obtained from a radial artery catheter at sea level every fifth minute during the hypoxic exposure and at the end of the 5 min’ walk. If the percutaneous oxygen saturation (SpO_2 HAST_) dropped ≤85% an extra arterial blood sample was taken. All samples were analysed immediately as previously described. The SpO_2 HAST_ was measured continuously with a finger probe (NONIN 8600, Medical, Inc., Minneapolis, USA). A 12-lead ECG monitoring (Cardiosoft, GE Marquette Medical Systems, Milwaukee, USA) was conducted throughout the test for evaluation of ischemia or arrhythmias. Dyspnoea was scored by the BORG CR10-scale [24].

### Statistical analysis

Demographic data are presented as mean values ± standard derivation (SD) unless otherwise specified. No missing values were imputed in the study. Differences between sea level values and HAST values were analysed using Student’s paired *T* test. Correlation between forced expiratory volume after 1 s (FEV_1_), PaO_2 SL_, SpO_2 SL_ and CPET variables at sea level and PaO_2 HAST_ were assessed by Pearson’s correlation coefficient (r) with 95% confidence intervals (CI). Statistical significance level was set to 5%.

## Results

Thirty-two patients (male = 21) were enrolled in the study to perform a HAST. Of these, 14 patients also performed a CPET. Two CF patients were excluded, one due to an on-going infection and one due to discomfort related to the artery catheter. The reason for declining to participate in the study was primarily due to refusal to accept arterial cannulation.

### Pre-flight values

Table 1 shows the patient demographics, spirometric values, resting sea level blood gas values, variables during CPET, and blood gas values during HAST. Fifteen patients (50%) had a FEV_1_ < 50% of predicted, 16 patients (53%) had a SpO_2 SL_ < 95%, and six patients (20%) had a PaO_2 SL_ < 9.3 kPa, cut-off values which have all been suggested as criteria for supplemental oxygen during air travel [3].

**Table 1:** Descriptive data of the participants, pulmonary function, blood gas variables and exercise variables, reported as mean (SD)

Age and anthropometry (n = 30)				
Age (yrs)	34.2 (11.81)			
Body mass (kg)	67.7 (16.35)			
Height (cm)	174.4 (9.31)			
BMI (kg/m^2^)	21.9 (3.65)			
Pulmonary function				
FEV_1_ (L)	2.25 (1.12)			
FEV_1_ (% predicted)	58.6 (24.8)			
FVC (L)	3.60 (1.22)			
FVC (% of predicted)	79.4 (20.9)			
FEV_1_/FVC ratio	60.5 (12.3)			

	Sea level	Exercise (n = 14)	HAST	HAST slow(n = 20) walking
Blood gas variables				
SpO_2_ (%)	94.8 (2.2)	88.6 (8.2)	88.6 (4.1)	87.0 (3.8)
SaO_2_ (%)	95.4 (1.5)	90.1 (8.4)	89.1 (2.8)	86.3 (3.8)
PaO_2_ (kPa)	10.2 (1.2)	9.8 (2.0)	7.4 (0.9)	6.9 (0.9)
PaCO_2_ (kPa)	5.0 (0.4)	–	5.0 (0.3)	5.0 (0.3)
Symptoms (BORG 10 CR)	0.09 (0.23)	–	0.5 (0.8)	0.9 (1.1)
CPET variables				
VE/VCO_2_		33.0 (4.9)		
VO_2max_ (ml·kg^−1^·min^−1^)		36.1 (10.5)		
VO_2max_ (% predicted)		87.5 (23.6)		
HR_max_ (beat·min^−1^)		175 (14.1)		

*BMI* body mass index, *FEV*_1_ forced expiratory volume in one second, *FVC* forced vital capacity, *SpO*_2_ saturation for oxygen measured by pulse oximetry, *VO*_*2max*_ maximal oxygen uptake, *HR*_*max*_ maximal heart rate during CPET

The average VO_2max_ during CPET (n = 14), was 36.1 ± 10.5 ml·kg^−1^·min^−1^, which was 88% of predicted. Four patients (29%) had a V_E_/VCO_2_ ratio >35, indicating ventilation/perfusion mismatching.

### Hypoxia altitude simulation test

In all patients, the PaO_2_ was considerably lower during HAST than at sea level (7.43 ± 0.89 vs 10.18 ± 1.19 kPa; p < 0.001). Four patients (13%) fulfilled the criteria for supplemental oxygen during air travel based on the BTS recommendations (PaO_2_ < 6.6 kPa) [3]. Of these, all had FEV_1_ < 50% of predicted, three had SpO_2 SL_ < 95%, and two had a PaO_2 SL_ < 9.3 kPa.

The average PaO_2 HAST_ during slow walking on the treadmill was 6.91 ± 0.87 kPa vs 7.55 ± 0.84 (p < 0.001) at rest (n = 27), adding another eleven patients to the group of subjects dropping below 6.6 kPa. Seven of these patients (23%) had FEV_1_ < 50%, five (17%) had SpO_2 SL_ < 95%, and two patients (7%) had a PaO_2 SL_ < 9.3 kPa.

No cardiac arrhythmias or signs of ischemia were observed during HAST. One patient reported moderate and two reported weak dyspnoea on the BORG CR10 scale. However, none of these patients fulfilled the criteria for supplemental oxygen during air travel.

### Correlation between pre-flight- and HAST variables

**Fig. 1:**
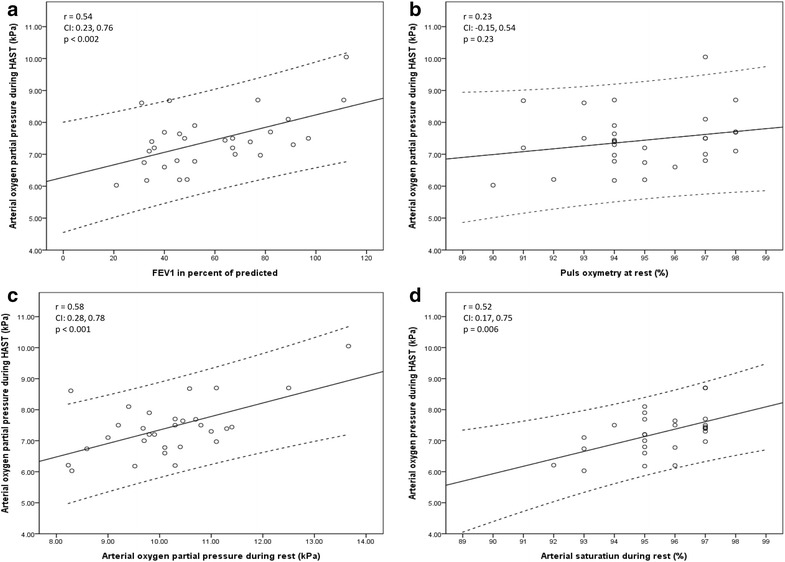
Relationships between arterial oxygen partial pressure (PaO_2_) during Hypoxia Altitude Simulation Test (HAST) and forced expiratory volume in one second (FEV_1_) (**a**), pulse oximetry (SpO_2_) (**b**), PaO_2_ (**c**) and SaO_2_ at rest (**d**)

**Fig. 2:**
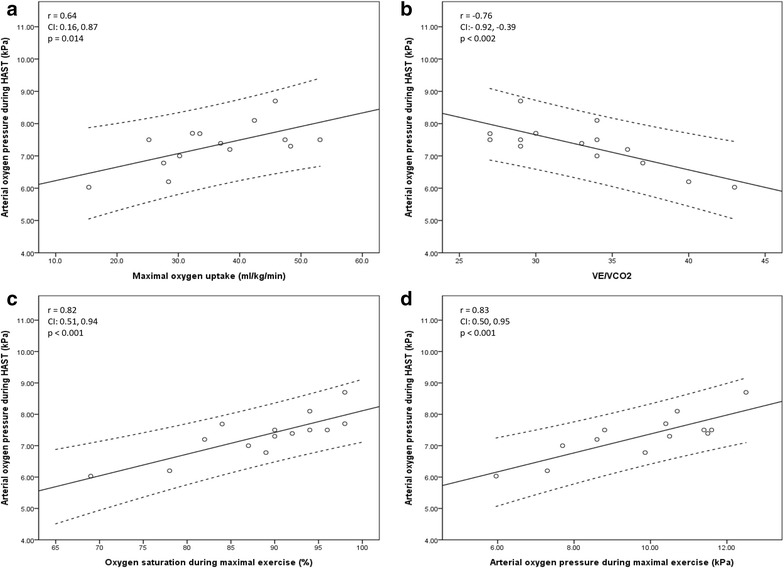
Relationships between arterial oxygen partial pressure (PaO_2_) during Hypoxia Altitude Simulation Test (HAST) and physiological variables during exercise; maximal oxygen uptake (**a**), ventilatory eqvivalent for carbon dioxide ratio (VE/VCO_2_) (**b**), pulse oximetry (SpO_2_) (**c**) and PaO_2_ (**d**)

Figure 1 shows the correlation between PaO_2 HAST_ and pre-flight values. There was a moderate correlation between PaO_2 HAST_ and FEV_1_, PaO_2 SL_ and SaO_2__SL_, while no significant correlation between PaO_2 HAST_ and SpO_2 SL_ was observed. Data from the CPET (Fig. 2) showed a strong correlation between PaO_2 HAST_ and PaO_2 CPET_, SpO_2 CPET_, VE/VCO_2 CPET_ and SaO_2 CPET_. In addition, a moderate correlation was observed between PaO_2 HAST_ and VO_2max_.

## Discussion

So far, no studies have demonstrated a simple and reliable method for identifying which CF-patients will become severely hypoxemic during air travel. In the present study, 30 CF patients were examined during a standardized hypoxia altitude simulation test. Four out of 30 patients dropped to a PaO_2_ < 6.6 kPa during HAST, thereby fulfilling an internationally accepted criterion for supplemental oxygen during air travel [3, 4]. In addition, 11 patients dropped below PaO_2_ < 6.6 kPa during slow walking on the treadmill while breathing the hypoxic gas mixture, underlining the impact of even very light physical activity during hypoxic conditions. PaO_2__HAST_ was modestly correlated to sea level FEV_1_, PaO_2_ and, SaO_2_ at rest. However, a strong correlation was observed between PaO_2__HAST_ and variables obtained during maximal exercise, i.e. PaO_2 CPET_, SpO_2 CPET_ and VO_2max_, and a strong negative correlation was observed between PaO_2 HAST_ and the V_E_/VCO_2_ ratio.

A methodological limitation of the study was that only 14 of the 30 patients performed a CPET. However, a post hoc power calculation for a correlation coefficient of 0.7 revealed that 13 participants were sufficient for detecting a significant difference from zero with 80% power.

### Pre-flight variables at rest

The usefulness of FEV_1_ in the pre-flight assessment has been debated. In a study of 69 adult CF patients, Peckham et al. found that FEV_1_ was significantly correlated to PaO_2 HAST_ [18]. In a study of 87 children with CF who travelled by air, Buchdahl et al. concluded that FEV_1_ was a better predictor of in-flight desaturation than HAST [25]. The results of the present study confirm a modest correlation between PaO_2 HAST_ and FEV_1_. The four CF-patients who fulfilled the criteria for supplemental oxygen all had a FEV_1_ below 50% of predicted, which is in agreement with other studies investigating adult CF-patients [17, 18]. It is, however, worth noting that 11 patients with a FEV_1_ < 50% predicted in the present study had a negative HAST (PaO_2_ ≥ 6.6 kPa). With regard to COPD patients, FEV_1_ has proven to be a poor predictor of in-flight hypoxaemia [10, 11]. The revised BTS recommendations regarding air travel state a poor relationship between FEV_1_ and hypoxaemia in passengers with pulmonary disease in general [3]. According to the literature, FEV_1_ seems to be a somewhat better predictor of in-flight hypoxaemia in CF patients than in patients with COPD, but both the present and previous studies are too small to draw reliable conclusions of whether CF patients with a FEV_1_ > 50% predicted can travel safely by air without supplementary oxygen.

Symptoms during HAST were in general mild in most patients, but increased slightly during slow walking. Only one of the patients that fulfilled the criteria for supplemental oxygen reported symptoms (weak), indicating that HAST was well tolerated in those who became very hypoxic.

Pulse oximetry is easy to perform and available at most medical centres. However, the latest BTS recommendations (2011) state that sea level SpO_2_ does not predict hypoxaemia as accurately as previously assumed [3]. This is in line with the current study showing that there was no significant correlation between pre-flight SpO_2_ and PaO_2 HAST_. One of the patients with a resting pulse oximetry ≥ 95% fulfilled the criteria for supplementary oxygen in the present study. Similar observations have been presented in a study on COPD patients [11]. Thus, a SpO_2_ ≥ 95% does not seem to be a reliable cut off value for travelling safely without supplemental oxygen. In a recently published study, an algorithm employing a combination of pulse oximetry at rest and during a 6 min walking test was used for pre-flight screening of COPD patients. It was found that this combination was a reliable tool for discriminating between COPD patients who can travel without supplementary oxygen, those who need such treatment, and the group of patients who needs more advanced pre-flight testing with HAST [26]. This algorithm has not been evaluated in relation to patients with CF.

In medical guidelines for air travel, it has been claimed that a stable pre-flight PaO_2_ exceeding 9.3 kPa is sufficient for travelling by air without supplemental oxygen [2, 5]. This has been contradicted by several studies on COPD patients [10, 11]. In the current study, two patients who required supplemental oxygen according to the HAST had a pre-flight PaO_2_ above 9.3 kPa (9.5 and 10.3 kPa). Hence, using pre-flight PaO_2_ > 9.3 kPa as a criterion for acceptable PaO_2_ levels during air travel in patients with CF does not exclude in-flight hypoxaemia.

### Pre-flight variables during exercise

The current study shows that PaO_2_ and SpO_2_ during maximal exercise are strongly correlated with PaO_2 HAST_. Also, the pre-flight VO_2max_ showed a stronger correlation to PaO_2 HAST_ than FEV_1_, SpO_2_ and PaO_2_ measured at rest. These findings are in agreement with previous findings in COPD patients during both hypobaric chamber exposure at 2438 m and during a commercial flight [10, 11, 27], showing that pre-flight aerobic capacity and exercise desaturation were strongly correlated with the in-flight PaO_2_. Since exercise testing is a part of the annual assessment at most CF-centres, exercise desaturation may be a good indicator for need of supplemental oxygen during air travel, also for CF patients with severe lung disease. In general, CPET is shown to be well tolerated in severely ill patients [28–30].

The ventilatory efficiency defined by ventilation relative to CO_2_-production (V_E_/VCO_2_) ratio [23] has not previously been studied as a variable in relation to development of in-flight hypoxaemia in patients with lung disease. In CF patients, hypoxaemia is mainly related to a mismatch between ventilation and perfusion in the lung caused by the chronic inflammation and mucus plugging [31], and an elevated V_E_/VCO_2_ ratio measured at the anaerobic threshold indicates an uneven distribution of ventilation and perfusion [23]. The V_E_/VCO_2_ ratio is highly reproducible, and the degree of elevation has been shown to reflect the severity of ventilation-perfusion mismatching [29]. The pre-flight V_E_/VCO_2_ ratio in the present study showed a strong negative correlation to PaO_2 HAST_. The two patients with the highest V_E_/VCO_2_ ratio both fulfilled the criteria for supplemental oxygen, and those patients who had a moderate elevation of V_E_/VCO_2_ ratio had a moderate reduction in PaO_2 HAST_. It should be noted that the V_E_/VCO_2_ ratio can also be elevated during hyperventilation due to anxiety or discomfort wearing a tight mask. However, no objective (high respiratory exchange ratio) or subjective sign of hyperventilation was observed during the test.

Taken together, these results indicate that exercise testing might be of value in the pre-flight assessment of lung patients, including patients with CF.

### Hypoxia altitude simulation test

HAST has proven to be a practical and reliable method for advanced pre-flight evaluation of patients with lung disease [32], and is today considered to be the ‘gold standard’ in clinical practise [26, 33]. In the present study, HAST has been used as the reference method for detecting development of hypoxaemia under hypoxic conditions. It should be noted, however, that Buchdahl et al. observed a poor relationship between SpO_2_ obtained during HAST and during a real flight [25]. This could be due to the fact that SpO_2_ seems to be inferior to PaO_2_ as a tool for predicting development of in-flight hypoxaemia [34]. As shown in other groups of lung patients, even light exercise seems to aggravate hypoxaemia under hypoxic conditions [10, 26]. This might be of importance for patients during long haul flights where the passengers are encouraged to exercise and also are in need of visiting the lavatory. Thus, in the authors’ opinion, light exercise should preferably be included in the HAST protocol. Other hazards than hypoxaemia during air travel may in general be the risk of venous thromboembolism due to immobilisation [35]. This condition may easily be prevented by use of compression stocking in combination with doing calf-muscle exercise. In-flight transmission of infectious disease is also a risk for CF patients [36].

## Conclusions

In summary, we have studied the relationship between the development of hypoxaemia during a simulated flight and regularly used tests of pulmonary function, blood gases and gas exchange variables during exercise in a group of adult patients with CF. Furthermore, the effect of slow walking during HAST was studied showing a pronounced aggravation of the hypoxaemia.

Variables obtained during CPET showed a stronger correlation to in-flight hypoxaemia than spirometric values and blood gas values obtained at rest. This is in accordance with studies on COPD patients. Thus, future studies should probably concentrate on establishing reliable cut off values during CPET, making them suitable for use in pre-flight screening of patients with CF.

## Abbreviations

BTS: British thoracic society
CF: cystic fibrosis
CI: confidence interval
COPD: chronic obstructive pulmonary disease
CPET: cardiopulmonary exercise test
FEV_1_: forced expiratory volume after one second
HAST: hypoxia-altitude simulation test
SL: sea level
SpO_2_: pulse oximetry
PaO_2_: arterial oxygen tension
V_E_: minute ventilation
VO_2_: oxygen uptake
VO_2max_: maximal oxygen uptake
